# Remarks on Insanity

**Published:** 1818-08

**Authors:** George Parkman

**Affiliations:** Boston, N. America.


### *For the London Medical and Physical Journal.*

*Remarks on Insanity; communicated* by George Parkman, M. D. of Boston, N. America.

DURING the Parliamentary Inquiry,* the increase of insanity in Great Britain, beyond that of population, was mentioned, without reference to the fact, that, when insane persons become objects of public attention, their number is better known. The same was mentioned in some parts of France, when asylums were enlarged or improved there, on account of the advantages of public over domestic management: also when an asylum near Philadelphia was proposed, 1810, and New York asylum, 1808. In Massachusetts 293 male, 256 female insane persons have come to my knowledge.

---

* This developed faults in neglected institutions. But the healing art applied to insanity, has ever furnished illustrious examples of men indefatigably, feelingly, and enlightenedly devoted to their toils, discouragements, responsibilities, privations, and perils. I here publicly express my obligations to that head of Psychological science, M. Pinel, to whom I was indebted for seven months' welcome access to the theatre of his labours, an asylum of 800 insane females, in Paris. His ripened years, 73, enjoy double experience in M. Esquirol, his colleague and former pupil, conductor of an excellent asylum for twenty persons. Ideas resulting from M. Pinel's experience gain additional force by passing before another intelligent, vigorous examiner. I might refer to numerous similar obligations, in performing a sort of promise I made in a letter to one of the Editors, published partly in the N. E. Med. Journal, Jan. 1813, and copied into the Lond. Med. and Phys. Journ. Jan. 1814. relating to a descriptive account of such asylums for insanity, in the British empire, France, Italy, and Switzerland, as are likely to offer themselves to the medical traveller's inspection. Outlines of this account, embracing 94 separate receptacles for insanity, were presented in manuscript to the trustees of the Gen. Hospital, before their asylum was planned.

### *Analogy between Sanity and Insanity.*

Some insane persons having exhibited wonderful changes from their ordinary state and conduct, insanity, according to the common notion, divests its subjects of the properties common to man, and represents them under states and ailments, opposite from our common nature. To these, specific management and medicine are thought necessary: till they are discovered, the subject is in a mystery thought to warrant the vaguest speculations. Consideration of the most striking extravagances of insanity in connexion with phenomena of sanity analogous to them, shows sanity and insanity are homogeneous, differing but in shades.* Many bereft of reason are like children not having it mature. Some are not unlike people fallen from eligible situations, who persist in notions fitted only to their former stations; *e. g.* heads of families, accustomed to command, are unwilling to consider themselves under direction. Aversion from society, desire of retirement, is seen in the sick, as in some insane persons. Agitating the fingers, feet, head, or body, muttering, humming, whistling, singing, biting the nails, among sane people, often seem analogous to the violent motion and raving of insanity, and resulting from a mind unfixed, or trying to divert itself from unpleasant objects.—A person in ill humour strikes his horse, dog, servant, or child; or is cross. Some insane persons, when an unpleasant idea enters their mind, outrage their attendant. In neither instance does there appear hostility to the aggrieved party, but uneasiness of a mind too insensible of relative duty. Hence the importance of invariably enforcing this duty.—Some insane persons see confusedly, like one half asleep, drunk, or dizzy; in reading, letters seem to run into each other.—Some invalids pick things from the street, and mortar from walls, and eat them; a singularity of appetite apparently analogous to that in which insane persons refuse common food, *et excrementa sua vorant.*

Inactivity of the will, and indecision, often exhibited by sane people, form principal points of treatment in melancholy. Dr. Gall told me of a mother, who said she felt an impulse to kill her children. She several times carried one of them to the river; momentary horror restrained her. She sometimes sharpened knives and put them under her pillow. Her husband, esteeming her a pious and affectionate mother, treated her anxiety lightly. Dr. G. advised her to absent herself from her family, as soon as she should first

* See also "Management of Lunatics, with illustrations of insanity by George Parkman, M. D." p. 6, 7.

feel these attacks. Some persons seem to feel involuntary impulse to steal even the most trifling and useless things. Daily other instances occur which, though not designated specimens of disordered intellect, no more than transient uneasiness, are called sickness; yet, though generally subsiding spontaneously, or without great curative means, may be the nucleus of great mental ailments; *e. g.* staying in bed late, when we wish ourselves out of bed, and make fruitless efforts to quit it: also at the fireside, at table, and in pleasant society. In anger we sometimes use expressions and actions disapproved by our experience and the looks of those about us. In high spirits we sometimes act so sillily and extravagantly, even friends, half amused, pleasantly call us light-headed, half-crazy. Then we say—resentment, or pleasant ideas overpowered us.—So insane persons sometimes say—I could not help it. They sometimes beg to be restrained, to supply self-control. In situations seeming partly bereft of motives to excite the will, see the idleness of the rich heir, the listlessness of the pauper who finds near him no means of employ immediately adequate to his need. A man without business paces his room to employ himself: he sees in the glass his face indicating a void of pleasing emotion. His unoccupied mind is ready for any impressions, and imagination is at leisure to modify them. Here seems to be the beginning of many cases of hypochondria, for the subsequent history of which see "Management of Lunatics," p. 12, &c.

*Suicide.*—Almost every body is occasionally indifferent of life. Should they then be in situations of great facilities for suicide, an attempt would not be surprising. People on an eminence feel strong propensity to leap down. Similar states appear in cattle, when their barns take fire; and in birds *charmed.* Suicide by poison, drowning, shooting, throat-cutting, sometimes occurs when the individual chances to meet with a poisonous substance, to be near the water, to be using arms for common purposes, or when he shaves; though a walk to the water, sporting, and shaving are often resorted to, to effect suicide, without exciting suspicion. The former suicides seem in a degree accidental, being, to premeditated ones, as manslaughter to murder. Some are purely accidental, as when a sleep-walker, or an insane person, leaps from a window, believing he is on the lower floor; these not having reason enough to foresee danger, may do most hazardous and fatal acts, without ill intent. Sir R. Croft was attending a protracted obstetric case, three months after his disappointment from the Princess Charlotte's death. In the room to which he had retired for repose, loaded pistols were hanging, with which he shot himself.

Coincidence of suicides is frequent, specially among friends and persons similarly situated. Knowledge of such acts seems too horrid for some persons' consideration; the impression so fixed, they kill themselves, apparently to be rid of it, as some convicts ask immediate execution, to save consideration of death; or as some patients, almost distracted in expectation of a surgical operation, are firm in suffering it; or as some ship-wrecked people leap into the sea, rather than view probable destruction. Werter determined to destroy himself to be rid of an idea he had conceived of murdering his friend and her husband. * * after the suicide of his bosom friend, being several times accused of attempting to follow his example, said—though the world presented to him but little to live for, his friend's suicide had so painfully affected him, there was no danger he should follow his example. He shot himself six months after his friend, holding in his hand a prayer for a person in the agonies of death. I know other analogous cases. Repeated suicides occurring in a family are often imputed to hereditary insanity, without reference to the circumstances above mentioned.

Of 95 suicides in and near Boston, 19 were committed by females.

Persons seeming disposed to suicide should be kept from objects likely to excite the disposition; manifestations of it are to be met by management, tending rather to divert it from its purpose than to thwart it. The following narrative I received from a physician to whom Dr. W. communicated it. The King proposed one day to shave *himself*. Dr. Willis feared to hesitate in assenting, lest he should seem to suspect intended suicide, and give the King dangerous notions, of the pre-existence of which there was no certainty. Dr. W. sent for the razors, and in the mean time engaged him about some papers on the table. The razors were put on the same table; he still attended to his papers, which encouraged Dr. W. to believe suicide was not intended. After shaving, the King returned to his papers; the razors were not removed immediately, lest Dr. W.'s anxiety about possible mischief should appear.

* * in mid-winter tried to leap into the neighbouring sea. As she passed, a tub of water thrown on her, producing a vivid impression, attracted her attention from her purpose, she ran back shivering, and never repeated such attempts. Cheerful scenes gave her only transient diversion; the contrast from them she found in her situation depressed her. I

showed her the interior of the alms-house; the distressed objects here convinced her she was not "the most wretched in the world," as she called herself. Few persons rightly value their advantages, not knowing others' sufferings. Most men think more of their troubles than if they knew others'. Cheerfulness often irritates melancholics, as singing and levity is offensive to some people occupied by thought.

Lord M. requested our distinguished artist G. Stuart, to paint a portrait of his brother, Captain C. P.: S. availed himself of much familiar intercourse with P. to witness in him those variations of countenance, which best exhibit characteristic animation.—M. saw the portrait, and exclaimed, "it is not my brother, it looks like an insane man!" In consequence, S. had another sitting; M. saw the portrait again, and exclaimed, "It is yet more like an insane man!" Three weeks after, P. shot himself. This, which might be thought impulse, seems a consummation of a mind in which disease had accumulated: insanity seemed to show its early stages.

*Sensibility and Insensibility to Heat and Cold.*—In some persons of great energy, vital heat seems superabundant. They wear no great coat, under-dress, gloves, or boots; sleep on a lightly-covered mattrass, open the window at rising, dress and undress in the cold, use cold bath in winter. Charles XII. of Sweden is said to have slept in mid-winter in Norway, in the open air, on straw, or a plank, covered only with a cloak. Another warrior is said to have harangued his army an hour in the streets of Warsaw, clad in white dimity. I know no insane persons' resistance to cold more wonderful than is exhibited by mariners on our coasts. Sometimes, in our coldest nights, a wave cases them with ice, when they dare not quit duty a moment. Their resistance seems to depend on strength, with mental energy excited by danger; in insanity, by a real or supposed state of things exciting energy. Security from cold seems to depend partly on the same mental principles, by which some persons are unhurt among apparent contagion.

Others, with the various preservatives of heat, are chilly, and pass the winter in trying to be warm. In some, insanity is so depressing, and inactivity so great, their susceptibility of cold is not strange. Hence the importance of encouraging activity in them. ****, æt. 17, had his toes frozen, a very mild night, middle of October, in a close room six feet square. He gave no previous intimation of suffering. Two others died, apparently chilled to death, October, a year before, in a warmer climate,—the weather very moderate. Some will not be clad, others inadvertently kick off the bedding. If crawling infants play as long as they please in parts of a room distant from the fire, in severe weather, their fingers, ears, and nose, will be half-frozen, without interrupting their pastime. Intermittent fevers present opposite extremes of temperature showing little connexion with atmospheric influence.

Some who have been insane and relieved never recover their force of mind, as some who have had bodily disease never recover their firmness. Those who have been insane are very susceptible to exciting causes, as those who have had bodily disease.

*** had an epileptic fit at a lecture in C. When it had subsided he knew not the place he was in, nor how he came there. Presently he remembered, in the order of occurrence, coming to C,—for a lecture,—entering the chapel,—the prayer,—text,—introduction of the lecture.

Painful dreams seem to result generally from baneful habits, ill health, or a mind disturbed when awake.

*Causes.*—In taking charge of insane persons, their friends' conjectures of the cause of the ailment have sometimes been given me. In 33 cases, physical causes have been mentioned; in 29, moral.

With the origin of each of at least 24 other cases, physical and moral circumstances seemed intimately connected.

At least 33 cases, imputed by friends to hereditary influence have been presented to me;

2 others combined with other or additional moral circumstances;

1 „ „ „ „ „ „ physical „

3 „ „ „ „ „ and moral „

Any exciting or depressing cause, acting on persons predisposed to insanity, may be followed by action of the predisposition. This remark seems applicable to some cases of insanity imputed to avarice, disappointed love, and religious enthusiasm.

Three insane persons have been committed to me, because their presence showed ill effect on some of their family. A person told the physician of an asylum,—with a near prospect of marriage, he thought of one or two instances of insanity among relations, he had painful doubts as to the propriety of fulfilling his marriage contract. He now inhabits that asylum, perhaps from dread of insanity, from painful scruples, from disappointment, from reproaches or suspicions of his intended relations, aggravated by maniacal predisposition. This is not a solitary case.

In ten cases insanity was imputed by friends to intemperance in strong drink.—In five others, before decided insanity, intemperance and other circumstances, physical and moral, co-existed, apparently conducive to insanity.—In three others, intemperance coexisted with moral circumstances, which seems frequent; intemperance destroys property and estimation, and begets disappointment, despair, &c.; strong drink is often resorted to, to exhilarate depressed minds.—In three others, hereditary and moral circumstance co-existed with intemperance.—In another, hereditary with intemperance.

Some persons, habitually temperate, previous to exciting suspicion of insanity show propensity to strong drink. Is not this an early *effect* of their insanity?

Some, after long intemperance, may be debarred from strong drink. Others may be supplied from a set of vessels, the capacity of which lessens in almost imperceptible gradation; or by diluting the draught; or by less stimulating sort of drink. I have satisfactorily substituted nice coffee, or strong hot mint-tea, or high-seasoned food. A child had got into use of opium at night. I occasionally lessened the opium a quarter, without his knowledge, and added rhubarb to preserve the accustomed size of his pills. He always discovered the difference.

Six cases imputed to religious enthusiasm have been presented to me: one of them combined with disappointed love; another with disappointed love and hereditary influence; another with other moral causes; another with physical causes.

Dr. Wistar, one of the Friends, told visitors to Pennsylvania Asylum,—insanity among the Friends seems often imputable to unduly active imagination, exerted about the divine spiritual faculty they specially believe communicated to men, independent of intellect. Inactivity of the will, or undue action of other mental principles, has been exemplified in convulsive epidemics among Methodists.

A person has been presented to me insane, apparently from "home-sickness." Those seem most exposed to it who have been accustomed to industry, temperance, domestic enjoyment, retirement, and scenes peculiar to their home. They generally try to hide the source of their uneasiness.

Insanity after bodily diseases seems often imputable to them; to anxiety as to their event; to affairs embarrassed, or supposedly embarrassed by them; to shame, when they rise from beginning vice; to premature return to accustomed occupations: all these, aggravated by susceptibility, increased by disease. Insanity after childbirth should be viewed in connexion with its sufferings; the changes consequent on sudden removal of distension and weight; new secretion and discharge, with their causes and consequences; sudden joy, relief from anxiety; sometimes disappointment and new anxieties; exertions induced by friends' congratulations. Of insanity apparently from more or less of these combined causes, at least thirteen cases have been presented to me.

A person with great irritability, impaired memory, unpleasant dreams, shame, doubt, discouragement, and sense of moral depravity, asked if his disease did not tend to weaken his intellect: if he had less sense than others? I asked as to his success in life. "He had prospered the last two years." I named to him one of his acquaintances, who had not prospered, yet thought well of himself. I concluded, apparently to his satisfaction, my patient had most sense of the two.

Many cases of insanity, imputed to intense study or bodily labour, occur under exertions conducted in ill health, prejudicially, or about objects perplexingly various, doubtful, or distant in event; then, study and labour show no necessary connexion with insanity; they generally seem highly preventive and curative of it.

Besides the general apparent agency of climate, and change of seasons in causing disease, lassitude opposed to salutary exertion attends people exposed to warm seasons.

The strongest evidence I know of certain changes in insane persons, at certain lunar periods, is in "*De l'Influence de la Nuit sur les Maladies*, 231—235. *a Bruxelles*, 1806."

To investigate thoroughly the causes of insanity, we must consider those pursuits of men which prevent full action of the intellect, also the general destitution of fixed principles, and the insubordination specially of the young to their natural guides. It may be necessary to fix a general period, styled the season of discretion; this ought not to be acknowledged when it proves false. As soon as an individual shows insensibility or disregard to the safeguards of his estimation or property, whatever be his age, it is the natural right and duty of those most interested in his welfare to seclude him from objects which apparently excited and maintain his delusion. If they scruple to do it, they are responsible for his consequent derangement or vice, the probable results of undirected minds impelled by excitement.—See also "Management of Lunatics," p. 30, l. 16, and p. 8—13.

*(To be concluded in our next.)*

